# Intratumor Heterogeneity in Early Lung Adenocarcinoma

**DOI:** 10.3389/fonc.2020.00349

**Published:** 2020-03-17

**Authors:** Maria-Fernanda Senosain, Pierre P. Massion

**Affiliations:** ^1^Division of Allergy, Pulmonary and Critical Care Medicine, Department of Medicine, Early Cancer Detection and Prevention Initiative, Vanderbilt Ingram Cancer Center, Vanderbilt University Medical Center, Nashville, TN, United States; ^2^Cancer Biology Graduate Program, Vanderbilt University, Nashville, TN, United States

**Keywords:** tumor behavior, molecular alterations, clonal evolution, single-cell, tumor microenvironment

## Abstract

Lung cancer is one of the deadliest diseases in the world and is the leading cause of cancer-related deaths. Among the histological types, adenocarcinoma is the most common, and it is characterized by a high degree of heterogeneity at many levels including clinical, behavioral, cellular and molecular. While most lung cancers are known for their aggressive behavior, up to 18.5% of lung cancers detected by CT screening are indolent and put patients at risk for overdiagnosis and overtreatment. The cellular and molecular underpinnings of tumor behavior remain largely unknown. In the recent years, the study of intratumor heterogeneity has become an attractive strategy to understand tumor progression. This review will summarize some of the current known determinants of lung adenocarcinoma behavior and discuss recent efforts to dissect its intratumor heterogeneity.

## Introduction

Over the last decades, several efforts have been made to reduce mortality among lung cancer patients. While advances in diagnostic and therapeutics have occurred, long-term survival rates compared to other cancers have barely improved (1). Therefore, new approaches are needed. In the context of lung adenocarcinoma (ADC), this is of great importance due to the high rate of overdiagnosis and lack of accuracy in predicting indolent vs. aggressive behavior of the tumor (2). In order to better predict disease behavior, it is crucial to understand the cellular and molecular underpinnings of the tumor. Thus, the study of intratumor heterogeneity and its clonal composition has become an attractive strategy to understand tumor progression and behavior (3–7). In the recent years emerging single-cell analysis platforms have allowed the deep profiling of the tumor microenvironment (TME), and seem promising approaches for the dissection and of tumor heterogeneity (8).

## An overview of lung ADC

Adenocarcinoma is a subclass of non-small cell lung cancer, which develops within the glandular cells of smaller airways along the outer edges of the lungs. It is the most common histological type, accounting for about 40% of all lung cancer cases. This type of lung cancer mostly occurs among current or former smokers, however it is also the most prevalent type of lung cancer in non-smokers (1). Thus, the exposure to environmental carcinogens combined with genetic susceptibility may also play an important role in the development of the disease (9, 10).

The survival rate for lung cancer mostly depends on the stage at the time of diagnosis. On average, the current 5-year survival rate is about 18%, but if detected early it can lead to a better prognosis, with a 5-year survival rate of 54% for localized stage (1). However, only 15% of all cases are diagnosed on time, while the vast majority (57%) are diagnosed at a late stage (11). Therefore, screening for lung cancer in high risk individuals is important.

In the past years, numerous randomized trials have assessed the power of lung cancer screening showing that it is possible to detect lung cancer at an early stage in more than 40% of the cases (12, 13). Furthermore, the 5- and 10-year survival rates among lung cancer patients enrolled in screening programs were close to 90%, which is very reassuring (14). The largest lung cancer screening trial at the moment, The National Lung Screening Trial (NLST), enrolled 53,452 high risk individuals for lung cancer across 33 U.S. medical centers and reported a 20% relative risk reduction in mortality using low-dose computed tomography (CT) screening compared to chest radiography (CXR) screening (15). Despite this encouraging statistics, it is worth to mention that 96% of the nodules detected through CT screening were benign. Moreover, confirmed lesions detected through CT screening range from very indolent to severely aggressive cancers. Therefore, screening, which by definition seeks to spot malignant nodules in asymptomatic individuals, bears the inherent feature of overdiagnosis. This phenomenon can be defined as the detection of a cancer that in other circumstances would have not become clinically evident, and represents a serious drawback for lung cancer screening in that it generates unnecessary treatment, morbidity, additional expenses, and anxiety and distress to the patient. A while after the NLST results were published, another study focused on the estimation of overdiagnosis in the NLST, reporting a probability of 18.5% that any lung cancer detected by LDCT was an overdiagnosis, as well as probabilities of 22.5% for non-small cell lung cancer and 78.9% for adenocarcinoma *in situ* (2). In that sense, a careful assessment of the images is crucial to ensure a more accurate prognosis. Additionally, the ongoing investigation in the discovery of new biomarkers offers a promising avenue to assist or eventually guide the screening and diagnosis process of high risk individuals.

## The Molecular Landscape of lung ADC

Over the years, genomic alterations occur and accumulate and in some cases those alterations may lead to oncogenesis. The somatic genomic alterations that are involved in cancer development are known as “driver alterations” and the ones that are not are known as “passenger alterations” (16). Lung ADC has one of the highest mutational burdens compared to other cancers (17, 18). Those high rates of somatic alterations and genomic rearrangements include a large load of passenger events per tumor genome, which makes the identification of driver alterations even more challenging (19). Despite the difficulties, several genomic alterations have been described in the past years, some of which are currently known as canonical driver alterations, and some others that have recently been reported and may be novel driver events (19–22).

Driver genomic alterations in lung ADC are generally associated with events that lead to the constitutive activation of signaling proteins, which commonly occur in oncogenes of the receptor tyrosine kinase (RTK)/RAS/RAF pathway (23). In the TCGA study, 62% of the tumors harbored such alterations (21). *KRAS* driver mutations were reported in 32% of TCGA samples (21). Along with *HRAS* and *NRAS* (0.9%), the other members of the RAS family, these proteins play an important role in the regulation of signaling pathways that control cell proliferation (24). Additionally, *KRAS* mutations are highly correlated with poor prognosis in early lung ADC (25). Cancer-associated mutations in *EGFR* were present in 11% of TCGA samples (21). *EGFR*, as well as other member of the EGFR family the oncogene *HER2* (1.7%), are known to be involved in the regulation of several cellular processes including cell motility, angiogenesis, cell proliferation and apoptosis (26). Likewise, some *EGFR* mutations are related to an improved prognosis (27). Another important oncogene is *BRAF*, which works downstream of RAS proteins and has a crucial role in the RAS-MAPK pathway. Driver mutations of this gene were present in 7% of TCGA samples and are not known to be associated with prognosis (21, 28). *MAP2K1* encodes for a protein that operates downstream of *BRAF* and was found mutated in 0.9% of TCGA samples (21). *MET* exon 14 skipping is another cancer driver event which results in the loss of a negative regulatory site, and occurred in 4.3% of TCGA samples (21). Gene fusions, were reported for the genes *ROS1, ALK*, and *RET*, which were altered in 1.7, 1.3, and 0.9% of TCGA samples, respectively (21, 23, 29).

In addition to the drivers described above, for the 38% of the samples that did not carry a driver oncogene mutation, the TCGA study proposed previously unrecognized driver genes that might be involved in the RTK/RAS/RAF pathway activation (21). They identified significant amplification events of *HER2* and *MET* in the oncogene-negative samples. Higher *MET* copy number in primary lung ADC at the time of diagnosis has been associated with poor prognosis (28). *NF1*, a tumor suppressor that negatively regulates the *RAS* oncogene, was mutated in 8.3% of the samples (21, 30). *RIT1* is mutated in 2.2% of ADC cases, and has been identified as a new oncogene driver as its mutations have been shown to activate MAPK and PI(3)K signaling in NIH3T3 cells (21, 31).

**Figure 1:**
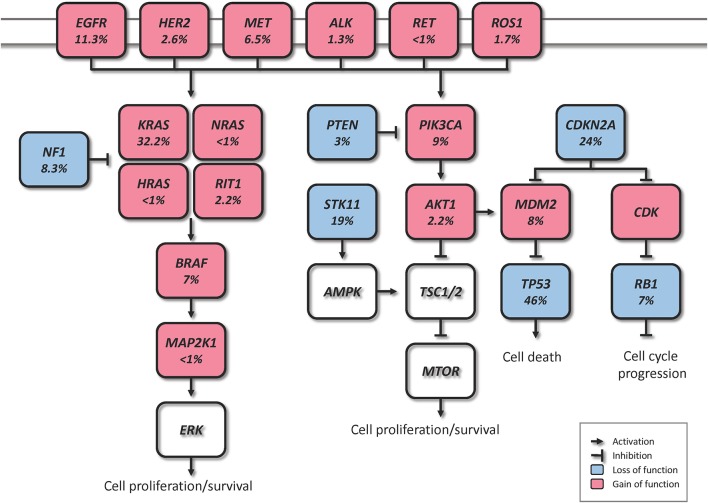
Canonical molecular pathways altered in lung ADC (% from TCGA).

Besides the RTK/RAS/RAF pathway, other relevant somatic genomic alterations have been identified. *TP53* was commonly mutated in 46% of the samples (21). *PIK3CA*, a crucial positive regulator of the PI(3)K-mTOR pathway, was mutated in 7% of the cases, and *STK11*, a tumor suppressor from the same pathway, was mutated in 17% of the cases (21). Other mutated tumor suppressors were *KEAP1* (17%), *RB1* (4%), and *CDKN2A* (4%). In a large-scale project that characterized copy-number alterations in lung ADC, the most common amplification was found in chromosome 14q13.3, which corresponds to NKX2-1 (TTF1), a transcription factor involved in lung development (20). The inhibition of this gene led to reduced cell viability and colony formation in lung ADC cell lines (20). This gene was also reported amplified in 14% of TCGA samples (21). Other significant amplifications in the TCGA study included the telomerase reverse transcriptase *TERT* (18%), and *MDM2* (8%), a negative regulator of p53 (21). The most significant deletion (19%) was the *CDKN2A* locus, which codes for the proteins p16 and p14arf, two important tumor suppressors and cell cycle regulators of the TP53 pathway (21, 32). Some of the alterations described above are depicted in Figure 1. The understanding of lung ADC molecular alterations has significantly impacted patient survival in the past years through the development of targeted therapies. Patients with advanced or metastatic tumors bearing *EGFR* mutations, *EML4-ALK* rearrangement or *ROS1* fusions have benefited from those. Erlotinib, gefitinib and afatinib are some of the drugs currently used to treat patients with *EGFR* exon 19 deletion or exon 21 mutations (33–35). Alectinib, ceritinib, and crizotinib have shown effectiveness in patients with *ALK* alterations, and the latter is also used in patients with *ROS1* translocation (36–39). The advances on genomic phenotyping of ADC have also benefited the development of immunotherapy. In a healthy individual, the immunecheckpoint PD-1 expressed in T cells protects against autoimmunity and inflammation. In cancer, PD-L1 expressed on tumor cells binds to PD-1 resulting in immunosupression and immune evasion. Nivolumab, pembrolizumab, and atezolizumab are some of the PD-1/PD-L1 FDA approved inhibitor drugs that have shown improved survival in advanced NSCLC patients compared to standard therapies (40–42). Another immunecheckpoint under the radar is CTLA-4. Two clinical trials (NCT02000947, NCT02352948) are currently investigating the effects of a combination therapy of dual checkpoint inhibition using durvalumab and tremelimumab, PD-1 and CTLA-4 inhibitors, respectively. However, early results suggest that this strategy did not significantly improved overall survival, although treatment with durvalumab alone provided a significant overall survival improvement (43, 44). These and other targeted therapies have been extensively reviewed previously (30, 45, 46).

More recently, the molecular characterization of early lung ADC lesions has also provided some insights on tumor behavior. A recent study from our group has characterized 21 adenocarcinoma *in situ* (AIS), 27 minimally invasive adenocarcinoma (MIA) and 54 fully invasive adenocarcinoma using deep targeted genome sequecing Qian et al. (47). This work uncovered molecular features associated with aggressive early ADC clinical behavior and disease progression. Most genomic alterations in ADC were already present in AIS and 21 significantly mutated genes including known drivers such as KRAS, EGFR and TP53 were shared among the three groups, suggesting their step-wise role in malignant transition. APOBEC signature was associated with worse survival compared to DNA mismatch repair signature, and KRAS codon 12 mutations were associated with aggressive tumor behavior. Finally, an ensemblelevel progression model using phylogenetic analysis inferred the role of many known alterations in lung ADC progression and introduced several new players such as EPPK1, ATM, SMAD4, KMT2C, and KMT2D, which deserve to be further investigated. This brings new insights into the distinction between indolent and aggressive tumor behavior and will potentially have future implications in early ADC clinical management.

## Intratumor Heterogeneity and Clonal Architecture

Intratumor heterogeneity is a highly complex phenomenon and it represents a major challenge in the assessment of cancer, as it acts as a confusing factor resulting in inaccurate diagnosis, prognosis and treatment of the disease (3). As mentioned before, lung ADC is a very heterogeneous disease with one of the highest mutational burdens across different cancer types (17, 18). Therefore, a comprehensive understanding of the natural history of these tumors is urgently needed.

The study of tumor growth from an evolutionary perspective is not a new approach. In the early 70's, Alfred Knudson proposed that for a particular cell to became cancerous, both alleles of a given tumor suppressor gene must be mutated, also known as the “two-hit hypothesis” Knudson (48). In 1976, Nowell applied evolutionary models to study tumor progression and treatment failure, and proposed a clonal evolution model in which a tumor arises from a single mutated cell (“clone”) and tumor progression occurs as a result of subsequent alterations, in which fitter and more aggressive clones replace the original clone cells (49). This linear evolution model was supported mostly by early studies that focused in a single gene rather than in the whole genome, and therefore clonal diversity was underestimated (50). Advances in new sequencing technologies allowed genome wide sequencing, which have elucidated a more complex clonal structure than previously thought (18).

In the past years, other evolutionary models have derived from applied phylogenetic inference to nextgeneration sequencing data. In neutral evolution, all driver alterations are thought to be present in the original neoplastic cell and subsequent alterations are neutral, thus it is characterized by the absence of selection and heterogeneity arises from stochastic processes as a byproduct of tumor progression (51). In punctuated evolution, it is postulated that tumor heterogeneity is generated in the early development of the neoplasia as a punctuated burst, followed by neutral evolution (52, 53). Branching evolution, also known as the trunkbranch model, is defined by the gradual accumulation of driver mutations in subclonal populations (54). In this model, the “trunk” of the tumor consists of progenitor clones bearing early somatic alterations that drive tumorigenesis. Those early alterations are potentially ubiquitous events. Conversely, somatic events that occur later are heterogeneous events and are present in the subclones which make up the “branches” of the tumor and are tumor progression drivers.

**Figure 2:**
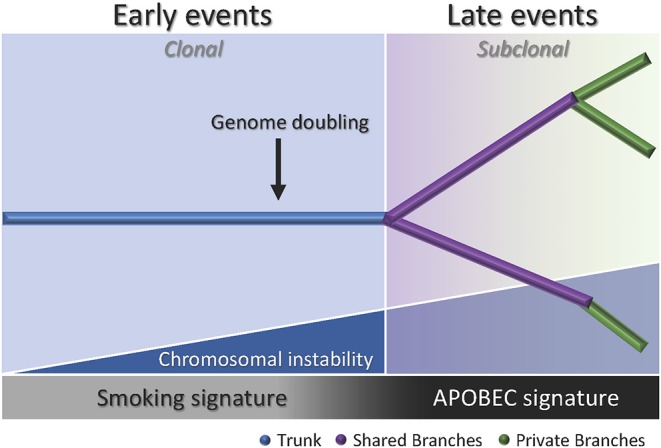
Branching process of tumor evolution in lung ADC. A tumor is depicted as a tree structure with the trunk representing ubiquitous (clonal) mutations present in all tumor regions (blue); shared branches representing heterogeneous (subclonal) mutations present in some tumor regions (purple), and private branches (also subclonal) representing unique mutations present in one tumor region only (green). The blue right triangle shows how as the chromosomal instability increases, the subclonal diversification is triggered. The bottom bar indicates that the smoking signature is associated with early events whereas the APOBEC signature is associated with late events.

Multiregion sequencing has been the most successful strategy to investigate intratumor heterogeneity and clonal evolution in lung ADC to date (4–6). The studies conducted by De Bruin and colleagues, Zhang and colleagues, and most recently Jamal-Hanjani and colleagues, provide evidence suggesting that intratumor heterogeneity and branched evolution might be a universal phenomenon across lung ADC (Figure 2). Most known driver alterations (21, 23) were mapped to the trunks of the tumors, which suggests that those canonical alterations occur early in tumor evolution. Truncal driver mutations almost always occurred before genome doubling suggesting a particular role in tumorigenesis. On the other hand, truncal genome doubling events occurred before subclonal diversification but after the acquisition of driver mutations, which suggests that chromosomal instability may be a crucial step that induces copy number alterations followed by a burst of mutational heterogeneity (Figure 2). Furthermore, the association of drug resistance and patient relapse with chromosomal instability (55), supports the hypothesis that the ability of chromosomal instability to generate extensive subclonal divergence could be compromising the effectiveness of therapeutics strategies that target truncal driver mutations due to the overlooked and already present clonal heterogeneity (4). Besides, data from these studies suggest that certain alterations in non-canonical cancer genes may also drive tumor development and subclonal diversification.

Another important feature of the disease addressed by these groups was the influence of smoking status in the clonal history of the tumors. Smoking signature (signature 4) is characterized by a high proportion of C>A transversions (18). In these studies, tumors from former and current smokers showed a decrease in the proportion of C>A transversions in subclonal mutations compared to early mutations, which suggests a relative decrease in the mutational burden due to smoking during tumor development (4–6). Moreover, the decrease of C>A transversions was followed by an increase in C>T and C>G mutations, which indicates APOBEC cytidine deaminase activity (18). This suggests that APOBEC mutagenesis may be playing a role in subclonal expansion in these tumors. In addition, a prolonged tumor latency period was reported by two groups (4, 6). In the study conducted by De Bruin and colleagues, a tumor from a patient that ceased smoking 20 years before surgery bore the smoking signature in more than 30% of truncal mutations, which suggests that these events occurred within a smoking tumorigenic setting more than 20 years ago (4). Likewise, Jamal-Hanjani and colleagues reported that 7 patients that were former smokers for several years before surgery, presented a smoking mutational signature suggesting tumor latency for several years before clinical manifestation of the disease (6). Furthermore, Zhang and colleagues and Jamal-Hanjani and colleagues found an association between the proportion of subclonal genomic alterations and recurrence (5, 6). In the cohort studied by first group, the three patients that relapsed had a significantly higher proportion of subclonal mutations compared to the patients with no relapse, suggesting that the degree of subclonal divergence may be associated with postsurgical relapse (5). In contrast, the second group did not find a significant association between the proportion of subclonal mutations and disease recurrence in their cohort, but found that patients with a large proportion of copy-number alterations were at higher risk for relapse or death compared to patients with a low proportion (6). Additionally, this group found that many late driver mutations corresponded to alterations that have been reported in other tumor types, and most of them are involved in genome maintenance processes such as DNA damage response, chromatin remodeling and histone methylation. They hypothesized that late mutations may be responsible for providing advantages to the emerging subclones and enabling the late stages of the disease as they may remove tissue specific constrains on the neoplastic genome (6).

These studies raised the question if single-region biopsy is informative enough to help the health providers make accurate treatment decisions. Intratumor heterogeneity has proven to be an intrinsic phenomenon to lung ADC, and it may compromise the ability of a single biopsy to comprehensively and accurately describe the complexity of the disease for an optimal cancer control. In a handful of cases, a large proportion of subclonal events were found in a single region but were absent in other regions of the same tumor, evidencing the limitations of a single-region biopsy in accurately explaining the clonal architecture of the tumor and highlighting the power of multiregion sequencing to better capture the clonality of the tumor which could help to prioritize some drug targets (4–6). Nonetheless, in the study conducted by Zhang et al., while they observed that multiregion sequencing is a better strategy to understand intratumor heterogeneity they also provided evidence that demonstrates that an increase in sequencing depth (277x to 863x) allowed the identification of most of the driver mutations in the tumors studied and many subclonal mutations were detectable in all regions of individual tumors. This suggests that a single biopsy analysis might be sufficient if the sequencing depth is increased (5).

## The Tumor Microenvironment of Lung ADC

It is known that the immune microenvironment plays a pivotal role in lung ADC development, thus it may also shape intratumor heterogeneity. Neoantigen presentation is an important step for cytolytic T cell response and it is guided by the human leukocyte antigen (HLA) class I molecule, which presents intracellular peptides on the cell surface for the T cell receptors to recognize (56). A person's genome contains up to six different HLA class I alleles encoded by the genes *HLAA, HLA-B* and *HLA-C*. Each HLA allotype presents peptide antigens based on specific anchor residues within the peptide sequence that are required for the peptides to bind. Therefore, loss of heterozygosity (LOH) results in loss of an HLA allotype and thus loss of the ability to bind those peptides that only contain anchor residues able to bind to the lost HLA molecule, hence fewer neoantigens can be presented to T cells. The impairment of tumor neoantigen presentation as a consequence of LOH in HLA class I was recently suggested as a mechanism of immune evasion in NSCLC (57). In this study, both lung adenocarcinomas and squamous cell carcinomas tumors with HLA LOH presented higher mutational burden compared to tumors without HLA LOH, with a significant increase in subclonal mutations. Furthermore, tumors harboring HLA LOH were enriched in neoantigens predicted to bind the missing HLA alleles and presented high PD-L1 staining on immune cells. This mechanism may facilitate the sub clonal expansion of cells harboring previously antigenic mutations that had become undetectable to the immune system. A following study from the same group, found that the immune microenvironment tends to be highly heterogeneous between and within patients, showing distinct regions with different levels of immune evasion within individual tumors (58). Additionally, tumors showing high immune infiltration and HLA allelic preservation also presented neoantigen depletion suggesting that immune evasion occurs by HLA LOH or neoantigen suppression. One of the possible mechanisms for the latter is promoter hypermethylation, which explains 23% of the neoantigens included in this study, suggesting that other mechanisms must be in place. Further elucidation of the mechanisms involved in neoantigen-associated immune escape could have important clinical implications in therapy selection and response prediction.

**Figure 3:**
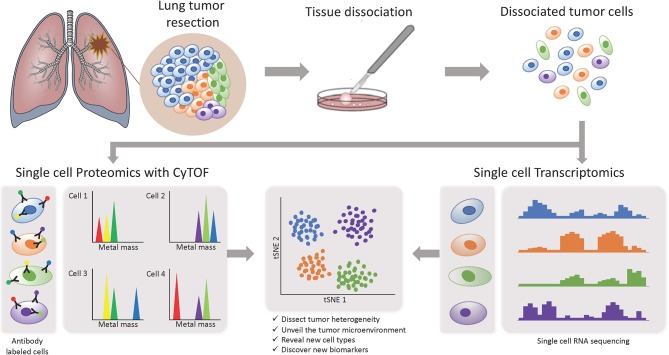
Investigating intratumor heterogeneity and the TME with single cell approaches. A lung tumor resection is dissociated into single cell suspension which can be used in different applications. CyTOF uses metal-labeled antibodies to detect a limited number of proteins in the cells. Single cell RNA-Seq reveals the transcriptome of each individual cell. Both can be analyzed through computational strategies to dissect intratumor heterogeneity.

In recent years, more studies focusing on the TME are starting to implement the use of single-cell based technologies, which can elucidate tumor heterogeneity with high resolution by detecting cells individually instead of a bulk signal and yield loads of information (Figure 3). Using single-cell proteomics mass cytometry analysis with paired tumor tissue, normal tissue and peripheral blood, Lavin and colleagues intended to provide an innate immune cell atlas of early LADC (59). In this study, early lesions have shown to bear a unique and TNM stage-independent immune signature, with a particular subset of tumor-infiltrating myeloid cells different from normal lung—PPARγ^hi^ macrophages enrichment and CD141+ dendritic cells (DC) depletion)—which could be compromising T cell immunity and may offer a new avenue of intervention in T cell immunotherapies. PPAR γ is a transcription factor known to drive an immunosuppressive program Reddy (60). Lymphotoxin beta, inflammatory response inducer, has been previously shown to act on high endothelial venules (HEV) to promote lymphocyte homing to peripheral lymph nodes *in vivo* Moussion and Girard (61). The authors found that the CD141+ DC subset expressed lymphotoxin beta transcripts in lung tumor tissues which suggests that CD141+ DC contribute to tertiary lymphoid structure formation likely through HEV-mediated recruitment of lymphocytes. Therefore, an induced expansion of intratumoral CD141+ DC may serve as a potential anti-tumor immunity strategy. This study highlights the importance of paired analysis to identify tumor-associated immune alterations from normal tissue-imprinting. Other study that also focused on tumor infiltrating myeloid cells (TIM), used single-cell RNA seq to profile a compare TIM populations between mice and humans in the context of NSCLC (62). Although the goal of this study was to establish similarities between mouse and human TIM expression programs, the comprehensive annotation of the different myeloid populations is an important contribution for future studies on clinical implications of the heterogeneity of these cell types. The authors reported that mouse and human TIM subsets show one-to-one equivalence and that blood myeloid cells poorly reflect TIM states. Due to the overlap of TIM states between patients they assessed the association with patient survival addressing the expression of genes specific to each subpopulation. They identified three conserved subsets of neutrophils, N1 that express canonical neutrophil markers, N2 which are tumor specific and promote tumor growth, and N2 which have a expression signature of type I interferon response. They found that human neutrophil subsets N2 and N5 showed an abundance of marker genes associated with poor survival. Conversely, the marker genes of human DC subset 2, which preferentially interacts with CD4+ T cells, showed positive association with survival. Guo and colleagues also investigated the immune system of NSCLC with single-cell RNA seq but focusing on T cell subpopulations of 14 patients (63). They identified two new CD8+ T cell pre-exhausted subsets, which together with the presence of highly migratory effector T cells may provide an explanation for positive responses to immunotherapy. When they interrogated LADC TGCA data with their expression signature, they found that patients mainly clustered into two groups: one enriched in pre-exhausted CD8+ T cells, non-activated Tregs and activated CD4+ T cells, and the other enriched in exhausted T cells and activated Tregs. Patients from group 1 had significantly better prognosis than patients from group 2, therefore T cell composition could be a potential clinical biomarker for LADC patients. In a different study, Lambrechts and colleagues used single-cell RNA sequencing and reported a comprehensive 52,698-cell catalog of the TME transcriptome of lung cancer samples, most of which were LADC patients (64). They identified 52 different stromal subtypes including different populations of cancer-associated fibroblasts, endothelial cells and infiltrating immune cells, some of which were further validated through immunofluorescence. Further analysis of TCGA data indicated that the abundances of some subpopulations and their correlation with patient survival differ between ADC and squamous cell carcinoma (SCC) and that they were influenced by clinical characteristics such as stage. Low expression of CD8 + T cell cluster 8 marker genes were positively and negatively associated with survival in ADC patients and SCC, respectively. This cluster represented CD8+ cytotoxic T cells per their high granzyme and IFN expression, and was characterized by high T cell exhaustion marker expression (LAG3). These and other gene expression changes in tumor stroma reveal potential new directions for intervention.

In conclusion, the TME represents an important component of tumor heterogeneity in LADC and is strongly associated with disease progression and predicted outcome. Although the different flavors of bulk profiling of the tumors are still providing a significant amount of information, it is important to acknowledge that singlecell approaches offer a new level of granularity that are allowing us to deeply dissect and further understand LADC heterogeneity and its implications in early stages of the disease. Nevertheless, such techniques are highly expensive which currently limits the number of samples per study. A combination of both bulk and single-cell approaches as reported in some of the studies mentioned above may be a suitable alternative to get the most out of the data while state-of-the-art techniques become more affordable through the years.

## Conclusions

Lung ADC is a devastating disease and despite the ongoing research efforts, the overall survival rates have barely improved in the past years. While screening programs have proven to significantly increase the chance of survival in high risk individuals, there is also a high probability of overdiagnosis. Therefore, the molecular determinants of early tumor development behavior need to be further investigated. In the past years, it has become more evident that intratumor heterogeneity profiling of lung ADC is the most effective strategy to understand tumor progression. In this context, the rapidly evolving field of single-cell technologies offers a novel set of tools that is unraveling the complexity of lung ADC and other cancers with a resolution never reached before.

## Author Contributions

M-FS wrote the manuscript with support from PM. PM conceived the original structure of the manuscript and supervised the project.

### Conflict of Interest

The authors declare that the research was conducted in the absence of any commercial or financial relationships that could be construed as a potential conflict of interest.

## References

[1] Siegel RL Miller KD Jemal A Cancer Statistics, 2016. J Clin. (2016) 66:7–30. 10.3322/caac.21332 26742998

[2] Patz EF Pinsky P Gatsonis C Sicks JD Kramer BS Tammemagi MC. Overdiagnosis in low-dose computed tomography screening for lung cancer. JAMA Int Med. (2014) 174:269. 10.1001/jamainternmed.2013.12738 24322569 PMC4040004

[3] Diaz-Cano SJ. Tumor heterogeneity: mechanisms and bases for a reliable application of molecular marker design. Int J Mol Sci. (2012) 13:1951–2011. 10.3390/ijms13021951 22408433 PMC3292002

[4] de Bruin EC McGranahan N Mitter R Salm M Wedge DC Yates L Spatial and temporal diversity in genomic instability processes deines lung cancer evolution. Science. (2014) 346:251–6. 10.1126/science.1253462 25301630 PMC4636050

[5] Zhang J Fujimoto J Zhang J Wedge DC Song X Zhang J. Intratumor heterogeneity in localized lung adenocarcinomas delinetated by multiregion sequencing. Science. (2014) 346:256–9. 10.1126/science.1256930 25301631 PMC4354858

[6] Jamal-Hanjani M Wilson GA McGranahan N Birkbak NJ Watkins TB Veeriah S Tracking the evolution of nonsmall-cell lung cancer. N Engl J Med. (2017) 376:2109–121. 10.1056/NEJMoa1616288 28445112

[7] Abbosh C Birkbak NJ Wilson GA Jamal-Hanjani M Constantin T Salari R. Phylogenetic ctDNA analysis depicts early-stage lung cancer evolution. Nature. (2017) 545:446–51. 10.1038/nature22364 28445469 PMC5812436

[8] Ortega MA Poirion O Zhu X Huang S Wolfgruber TK Sebra R. Using single-cell multiple omics approaches to resolve tumor heterogeneity. Clin Transl Med. (2017) 6:46. 10.1186/s40169-017-0177-y 29285690 PMC5746494

[9] Gibelin C Couraud S. Somatic alterations in lung cancer: do environmental factors matter? Lung Cancer. (2016) 100:45–52. 10.1016/j.lungcan.2016.07.015 27597280

[10] Rivera GA Wakelee H. Lung Cancer in Never Smokers. Cham: Springer International Publishing.

[11] Torre LA Siegel RL Jemal A. Lung Cancer Statistics. Cham: Springer International Publishing.

[12] van Klaveren RJ Oudkerk M Prokop M Scholten ET Nackaerts K Vernhout R. Management of lung nodules detected by volume CT scanning. N Engl J Med. (2009) 361:2221–9. 10.1056/NEJMoa0906085 19955524

[13] Blanchon T Bre'chot JM Grenier PA Ferretti GR Lemarie' E Milleron B. Baseline results of the Depiscan study: a French randomized pilot trial of lung cancer screening comparing low dose CT scan (LDCT) and chest X-ray (CXR). Lung Cancer. (2007) 58:50–8. 10.1016/j.lungcan.2007.05.009 17624475

[14] Vinet L Zhedanov A Survival of patients with stage I lung cancer detected on CT screening. N Engl J Med. (2006) 355:1763–71. 10.1056/NEJMoa060476 17065637

[15] National Lung Screening Trial Research Team Aberle DR Adams AM Berg CD Black WC Clapp JD. Reduced lung-cancer mortality with low-dose computed tomographic screening. N Engl J Med. (2011) 365:395–409. 10.1056/NEJMoa1102873 21714641 PMC4356534

[16] Stratton MR Campbell PJ Futreal PA. The cancer genome. Nature. (2009) 458:719–24. 10.1038/nature07943 19360079 PMC2821689

[17] Kandoth C McLellan MD Vandin F Ye K Niu B Lu C. Mutational landscape and significance across 12 major cancer types. Nature. (2013) 502:333–9. 10.1038/nature12634 24132290 PMC3927368

[18] Alexandrov LB Nik-Zainal S Wedge DC Aparicio SAJR Behjati S Biankin AV. Signatures of mutational processes in human cancer. Nature. (2013) 500:415–21. 23945592 10.1038/nature12477 PMC3776390

[19] Imielinski M Berger AH Hammerman PS Hernandez B Pugh TJ Hodis E. Mapping the hallmarks of lung adenocarcinoma with massively parallel sequencing. Cell. (2012) 150:1107–20. 10.1016/j.cell.2012.08.029 22980975 PMC3557932

[20] Weir BA Woo MS Getz G Perner S Ding L Beroukhim R. Characterizing the cancer genome in lung adenocarcinoma. Nature. (2007) 450:893–8. 10.1038/nature06358 17982442 PMC2538683

[21] Collisson EA Campbell JD Brooks AN Berger AH Lee W Chmielecki J Comprehensive molecular profiling of lung adenocarcinoma. Nature. (2014) 511:543–50. 10.1038/nature13385 25079552 PMC4231481

[22] Berger AH Brooks AN Wu X Shrestha Y Chouinard C Piccioni F. High-throughput phenotyping of lung cancer somatic mutations. Cancer Cell. (2016) 30:214–28. 10.1016/j.ccell.2016.06.022 27478040 PMC5003022

[23] Pao W Hutchinson KE. Chipping away at the lung cancer genome. Nat Med. (2012) 18:349–51. 10.1038/nm.2697 22395697

[24] Downward J. Targeting RAS signalling pathways in cancer therapy. Nat Rev Cancer. (2003) 3:11–22. 10.1038/nrc969 12509763

[25] Kadota K Sima CS Arcila ME Hedvat C Kris MG Jones DR. KRAS mutation is a significant prognostic factor in early-stage lung adenocarcinoma. Am J Surg Pathol. (2016) 40:1579–90. 10.1097/PAS.0000000000000744 27740967 PMC5106330

[26] Wieduwilt MJ Moasser MM. The epidermal growth factor receptor family: biology driving targeted therapeutics. Cell Mol Life Sci. (2008) 65:1566–84. 10.1007/s00018-008-7440-8 18259690 PMC3060045

[27] Liu WS Zhao LJ Pang QS Yuan ZY Li B Wang P. Prognostic value of epidermal growth factor receptor mutations in resected lung adenocarcinomas. Med Oncol. (2014) 31:771. 10.1007/s12032-013-0771-9 24248816

[28] Sholl LM. Biomarkers in lung adenocarcinoma: a decade of progress. Arch Pathol Lab Med. (2015) 139:469–80. 10.5858/arpa.2014-0128-RA 25255293

[29] Bergethon K Shaw AT Ou SHI Katayama R Lovly CM McDonald NT. ROS1 rearrangements define a unique molecular class of lung cancers. J Clin Oncol. (2012) 30:863–70. 10.1016/j.yonc.2012.06.023 22215748 PMC3295572

[30] Chalela R Curull V Enr'iquez C Pijuan L Bellosillo B Gea J. Lung adenocarcinoma: from molecular basis to genome-guided therapy and immunotherapy. J Thoracic Dis. (2017) 9:2142–58. 10.21037/jtd.2017.06.20 28840016 PMC5542927

[31] Berger AH Imielinski M Duke F Wala J Kaplan N Shi GX. Oncogenic RIT1 mutations in lung adenocarcinoma. Oncogene. (2014) 33:4418–23. 10.1038/onc.2013.581 24469055 PMC4150988

[32] De Snoo FA Hayward NK. Cutaneous melanoma susceptibility and progression genes. Cancer Lett. (2005) 230:153–86. 10.1016/j.canlet.2004.12.033 16297704

[33] Wu YL Zhou C Liam CK Wu G Liu X Zhong Z First-line erlotinib versus gemcitabine/cisplatin in patients with advanced EGFR mutation-positive non-small-cell lung cancer: analyses from the phase III, randomized, openlabel, ENSURE study. Ann Oncol. (2015) 26:1883–9. 10.1093/annonc/mdv270 26105600

[34] Mitsudomi T Morita S Yatabe Y Negoro S Okamoto I Tsurutani J. Gefitinib versus cisplatin plus docetaxel in patients with non-small-cell lung cancer harbouring mutations of the epidermal growth factor receptor (WJTOG3405): an open label, randomised phase 3 trial. Lancet Oncol. (2010) 11:121–8. 10.1016/S1470-2045(09)70364-X 20022809

[35] Sequist LV Yang JCH Yamamoto N O'Byrne K Hirsh V Mok T Phase III study of afatinib or cisplatin plus pemetrexed in patients with metastatic lung adenocarcinoma with EGFR mutations. J Clin Oncol. (2013) 31:3327–34. 10.1200/JCO.2012.44.2806 23816960

[36] Shaw AT Gandhi L Gadgeel S Riely GJ Cetnar J West H. Alectinib in ALK-positive, crizotinib-resistant, non-small-cell lung cancer: a single-group, multicentre, phase 2 trial. Lancet Oncol. (2016) 17:234–42. 10.1016/S1470-2045(15)00488-X 26708155 PMC4752892

[37] Shaw AT Kim DW Mehra R Tan DS Felip E Chow LQ Ceritinib in ALK-rearranged non-small-cell lung cancer. N. Engl. J. Med. (2014) 370:1189–97. 10.1056/NEJMoa1311107 24670165 PMC4079055

[38] Chuang JC Neal JW. Crizotinib as first line therapy for advanced ALK-positive non-small cell lung cancers. Transl Lung Cancer Res. (2015) 4:639–41. 10.3978/j.issn.2218-6751.2015.03.06 26629437 PMC4630528

[39] Shaw AT Ou SHI Bang YJ Camidge DR Solomon BJ Salgia R Crizotinib in ROS1-rearranged nonsmall-cell lung cancer. N Engl J Med. (2014) 371:1963–71. 10.1093/annonc/mdu349.78 25264305 PMC4264527

[40] Borghaei H Paz-Ares L Horn L Spigel DR Steins M Ready NE. Nivolumab versus docetaxel in advanced nonsquamous non-small-cell lung cancer. N Engl J Med. (2015) 373:1627–39. 10.1056/NEJMoa1507643 26412456 PMC5705936

[41] Herbst RS Baas P Kim DW Felip E P e'rez-Gracia JL Han JY. Pembrolizumab versus docetaxel for previously treated, PD-L1-positive, advanced non-small-cell lung cancer (KEYNOTE-010): a randomised controlled trial. Lancet. (2016) 387:1540–50. 10.1016/S0140-6736(15)01281-7 26712084

[42] Rittmeyer A Barlesi F Waterkamp D Park K Ciardiello F von Pawel J. Atezolizumab versus docetaxel in patients with previously treated non-small-cell lung cancer (OAK): a phase 3, open-label, multicentre randomised controlled trial. Lancet. (2017) 389:255–65. 10.1016/S0140-6736(16)32517-X 27979383 PMC6886121

[43] Garon EB Spira AI Goldberg SB Chaft JE Papadimitrakopoulou V Antonia SJ Safety and activity of durvalumab + tremelimumab in immunotherapy (imt) pretreated advanced nsclc patients. J Clin Oncol. (2018) 36:9041 10.1200/JCO.2018.36.15_suppl.9041

[44] Kowalski DM Orlov SV Fischer JR Medine DM Soo RA Geater SL. Arctic: Durvalumab + Tremelimumab and durvalumab monotherapy vs SOC in 3l advanced NSCLC treatment. Ann Oncol. (2018) 29(Suppl. 8):viii493–viii547. 10.1093/annonc/mdy292.001

[45] Parums DV. Current status of targeted therapy in non-small cell lung cancer. Drugs Today. (2014) 50:503–25. 10.1358/dot.2014.50.07.2185913 25101332

[46] Hirsch FR Scagliotti GV Mulshine JL Kwon R Curran WJ Wu YL. Lung cancer: current therapies and new targeted treatments. Lancet. (2017) 389:299–311. 10.1016/S0140-6736(16)30958-8 27574741

[47] Qian J Zhao S Zou Y Rahman SJ Senosain M-F Stricker T. Genomic underpinnings of tumor behavior in *in situ* and early lung adenocarcinoma. Am J Respirat Crit Care Med. (2019). 10.1164/rccm.201902-0294OC. [Epub ahead of print]. 31747302 PMC7068818

[48] Knudson AG. Mutation and cancer: statistical study of retinoblastoma. Proc Natl Acad Sci USA. (1971) 68:820–3. 10.1073/pnas.68.4.820 5279523 PMC389051

[49] Nowell PC Nowell PC. The clonal evolution of tumor cell populations. Science. (1976) 194:23–8. 10.1126/science.959840 959840

[50] Swanton C. Intratumor heterogeneity: evolution through space and time. Cancer Res. (2012) 72:4875–82. 10.1158/0008-5472.CAN-12-2217 23002210 PMC3712191

[51] Williams MJ Werner B Barnes CP Graham TA Sottoriva A. Identification of neutral tumor evolution across cancer types. Nat Genet. (2016) 48:238–44. 10.1038/ng.3489 26780609 PMC4934603

[52] Baca SC Prandi D Lawrence MS Mosquera JM Romanel A Drier Y. Punctuated evolution of prostate cancer genomes. Cell. (2013) 153:666–77. 10.1016/j.cell.2013.03.021 23622249 PMC3690918

[53] Sottoriva A Kang H Ma Z Graham TA Salomon MP Zhao J. A big bang model of human colorectal tumor growth. Nat Genet. (2015) 47:209–16. 10.1038/ng.3214 25665006 PMC4575589

[54] Gerlinger M Rowan AJ Horswell S Larkin J Endesfelder D Gronroos E. Intratumor heterogeneity and branched evolution revealed by multiregion sequencing. N Engl J Med. (2012) 366:883–92. 10.1056/NEJMoa1113205 22397650 PMC4878653

[55] Lee AJ Endesfelder D Rowan AJ Walther A Birkbak NJ Futreal PA. Chromosomal instability confers intrinsic multidrug resistance. Cancer Res. (2011) 71:1858–70. 10.1158/0008-5472.CAN-10-3604 21363922 PMC3059493

[56] Cruz-Tapias P Castiblanco J Anaya J-M Chapter 10: Major histocompatibility complex: Antigen processing and presentation. In: Anaya JM Shoenfeld Y Rojas-Villarraga A editors. Autoimmunity: From Bench to Bedside. Bogota: El Rosario University Press (2013). 29087650

[57] McGranahan N Rosenthal R Hiley CT Rowan AJ Watkins TB Wilson GA. Allele-specific HLA loss and immune escape in lung cancer evolution. Cell. (2017) 171:1259–71.e11. 10.1016/j.cell.2017.10.001 29107330 PMC5720478

[58] Rosenthal R Cadieux EL Salgado R Bakir MA Moore DA Hiley CT. Neoantigen-directed immune escape in lung cancer evolution. Nature. (2019) 567:479–85. 10.1038/s41586-019-1032-7 30894752 PMC6954100

[59] Yonit Lavin A Kobayashi S Leader A Rahman A Amit I Merad Correspondence M. Innate Immune Landscape in Early Lung Adenocarcinoma by Paired Single-Cell Analyses. Cell. (2017) 169:750–7.e15. 10.1016/j.cell.2017.04.014 28475900 PMC5737939

[60] Reddy RC. Immunomodulatory role of PPAR-γ in alveolar macrophages. J Invest Med. (2008) 56:522–7. 10.2310/JIM.0b013e3181659972 18317435

[61] Moussion C Girard JP. Dendritic cells control lymphocyte entry to lymph nodes through high endothelial venules. Nature. (2011) 479:542–6. 10.1038/nature10540 22080953

[62] Zilionis R Engblom C Pfirschke C Savova V Zemmour D Saatcioglu HD. Single-cell transcriptomics of human and mouse lung cancers reveals conserved myeloid populations across individuals and species. Immunity. (2019) 50:1317–34.e10. 10.1016/j.immuni.2019.03.009 30979687 PMC6620049

[63] Guo X Zhang Y Zheng L Zheng C Song J Zhang Q Global characterization of T cells in non-small-cell lung cancer by single-cell sequencing. Nat Med. (2018) 24:978–85. 10.1038/s41591-018-0045-3 29942094

[64] Lambrechts D Wauters E Boeckx B Aibar S Nittner D Burton O. Phenotype molding of stromal cells in the lung tumor microenvironment. Nat Med. (2018) 24:1277–89. 10.1038/s41591-018-0096-5 29988129

